# Biochemical, Transcriptional, and Bioinformatic Analysis of Lipid Droplets from Seeds of Date Palm (*Phoenix dactylifera* L.) and Their Use as Potent Sequestration Agents against the Toxic Pollutant, 2,3,7,8-Tetrachlorinated Dibenzo-*p*-Dioxin

**DOI:** 10.3389/fpls.2016.00836

**Published:** 2016-06-08

**Authors:** Abdulsamie Hanano, Ibrahem Almousally, Mouhnad Shaban, Farzana Rahman, Elizabeth Blee, Denis J. Murphy

**Affiliations:** ^1^Department of Molecular Biology and Biotechnology, Atomic Energy Commission of SyriaDamascus, Syria; ^2^Genomics and Computational Biology Group, University of South WalesPontypridd, UK; ^3^Institut de Biologie Moléculaire des PlantesStrasbourg, France

**Keywords:** date palm, *Phoenix dactylifera*, lipid droplets, oleosins-like, sequestration, TCDD, dioxins

## Abstract

Contamination of aquatic environments with dioxins, the most toxic group of persistent organic pollutants (POPs), is a major ecological issue. Dioxins are highly lipophilic and bioaccumulate in fatty tissues of marine organisms used for seafood where they constitute a potential risk for human health. Lipid droplets (LDs) purified from date palm, *Phoenix dactylifera*, seeds were characterized and their capacity to extract dioxins from aquatic systems was assessed. The bioaffinity of date palm LDs toward 2,3,7,8-tetrachlorodibenzo-*p*-dioxin (TCDD), the most toxic congener of dioxins was determined. Fractioned LDs were spheroidal with mean diameters of 2.5 µm, enclosing an oil-rich core of 392.5 mg mL^-1^. Isolated LDs did not aggregate and/or coalesce unless placed in acidic media and were strongly associated with three major groups of polypeptides of relative mass 32–37, 20–24, and 16–18 kDa. These masses correspond to the LD-associated proteins, oleosins, caleosins, and steroleosins, respectively. Efficient partitioning of TCDD into LDs occurred with a coefficient of log *K*_LB/w,TCDD_ = 7.528 ± 0.024; it was optimal at neutral pH and was dependent on the presence of the oil-rich core, but was independent of the presence of LD-associated proteins. Bioinformatic analysis of the date palm genome revealed nine oleosin-like, five caleosin-like, and five steroleosin-like sequences, with predicted structures having putative lipid-binding domains that match their LD stabilizing roles and use as bio-based encapsulation systems. Transcriptomic analysis of date palm seedlings exposed to TCDD showed strong up-regulation of several caleosin and steroleosin genes, consistent with increased LD formation. The results suggest that the plant LDs could be used in ecological remediation strategies to remove POPs from aquatic environments. Recent reports suggest that several fungal and algal species also use LDs to sequester both external and internally derived hydrophobic toxins, which indicates that our approach could be used as a broader biomimetic strategy for toxin removal.

## Introduction

Plant LDs, also known as lipid bodies, oil bodies, or oleosomes, are cytosolic organelles in which plants store their major oil reserves (Murphy, 2012). Such LDs are most commonly found in oil-rich seeds, in oil-storing pollen grains, and in the mesocarp tissues of oily fruits such as avocado, olive, and oil palm (Huang, 1992; Murphy, 1993). The LDs in higher plant seeds are typically made up of a TAG core surrounded by a PL monolayer with a characteristic population of embedded proteins, the most abundant of which are oleosins, caleosins, and steroleosins with approximate molecular weight ranges of 17–20, 22–24, and 32–37 kDa, respectively (Tzen et al., 1993). These proteins confer a remarkable stability that prevents LD aggregation or coalescence over a wide range of environmental conditions. In the case of oilseeds, their LDs remain as intact emulsions after extreme desiccation during seed development and subsequent post-germinative rehydration (Beisson et al., 2001) and during prolonged storage for months or even decades or longer (Leprince et al., 1998).

Hydrophobic organic compounds are reported to show differential affinities toward TAGs or PL (Sandermann, 2003). Increasingly hydrophobic compounds (log *K*_ow_ > 3) show a higher affinity for TAG and vice-versa. In this context, Boucher et al. (2008) have demonstrated that isolated LDs from rapeseed were capable of effective extraction of some hydrophobic pollutants such as atrazine, carbaryl, parathion, naphthalene, and 2-phenylethanol from aqueous environments. The authors showed that such compounds were absorbed into the inner oily core of the LDs and that the monolayer membrane did not represent a barrier for the mass transfer of the compounds toward this core. It has been suggested that, due to their high surface/volume ratios, plant LDs may exhibit superior mass transfer properties compared with larger synthetic microcapsules or two-phase LLE systems (Boucher et al., 2008).

The plant model for the present study, date palm (*Phoenix dactylifera* L.), is a perennial monocotyledonous species that is native to the Middle East and North Africa. The date palm is believed to have originated from the region around the Arabian Gulf and has long been regarded as a prestigious tree. Date palms are well-acclimatized to desert environments and especially thrive in the dry sandy soils that are so common in this region (Morton, 1987; Chao and Krueger, 2007). The sequencing of the date palm genome was announced in 2012 and these data are now available in public repositories (Al-Mssallem et al., 2013).

For economic, ecological and genetic reasons, date palm trees are typically regenerated using *in vitro* somatic embryogenesis. The large numbers of seeds produced by the date palm industry are considered as by-products that are either discarded or used in low value commodities such as animal feed. However, previous chemical analysis showed that date palm seeds contain 8–10% w/w oil (Besbes et al., 2004). Equal amounts of saturated and unsaturated (mostly monounsaturated) fatty acids are present in date palm seed oil and the major components are lauric (C12:0), palmitic (C16:0), stearic (C18:0), oleic (C18:1), and linoleic (C18:2) acids (Basuny and Al-Marzooq, 2011; Abdalla et al., 2012). This acyl composition gives date palm oil high stability over a wide temperature range making it suitable for the extracted LDs to be used for *in vitro* applications in a variety of external environments.

Collectively termed dioxins, PCDDs and PCDFs are notorious as one of the most toxic group of POPs. Consisting of two aromatic rings linked via one (PCDFs) or two (PCDDs) atoms of oxygen and 1–8 fixed chlorine atoms, these halogenated chemicals are structurally very stable and hydrophobic but also capable of accumulating in aqueous media. These characteristics enable dioxins to persist in the environment and bioaccumulate in food chains (Pollitt, 1999). Contamination of the aquatic environment with dioxins is considered one of the major ways by which these chemicals can be transmitted between aquatic organisms and go on to bioaccumulate in many types of seafood (Tsutsumi et al., 2003). This can lead to exposure of humans to dioxins via contaminated seafood as well as from direct contact with these toxic agents (Baeyens et al., 2007).

An effective way stopping of the biotransmission of dioxins in aquatic ecosystems requires an early intervention that can guarantee safe and efficient removal of such pollutants at or close to the time of their release. Several previous strategies have been proposed to remove dioxins and other organic pollutants from aquatic environments based on their high lipophilicity (Wyss et al., 2004). For example, Watarai suggested that emulsions or micelles with an oily core could be used to extract lipophilic molecules from aqueous systems. However, these micelles tended to be relatively unstable and to coalesce, particularly when agitated (Watarai, 1997). As an alternative, the use of polymerisable surfactants (surfmers) to stabilize oil-core micelles has been proposed (Summers and Eastoe, 2002). Interestingly, the stabilization of oil-core capsules with proteins has been successfully achieved (Suslick and Grinstaff, 1990; Makino et al., 2002). In particular, lipid-binding oleosins from plant LDs have been investigated as lipid-encapsulating agents since they contain a lengthy hydrophobic central domain. This domain, which is one of the longest continuous hydrophobic sequences found in any naturally occurring protein described to date, acts as a highly effective lipid anchor that emulsifies the LDs into small spheroidal droplets in the range of 1–3 μm and greatly increases droplet stability both *in vivo* and *in vitro* (Huang, 1996).

Plant LDs have been investigated as possible carriers of lipophilic molecules for biotechnological applications such as the overexpression of valuable compounds and can also act as sites for the bioaccumulation of signaling molecules such as phytoalexins (Bonsegna et al., 2011; Shimada et al., 2014). However, the use of isolated plant LDs for extracting hydrophobic compounds from aqueous environments is a relatively unexplored area of research. To date there has been a single report on the use of rapeseed LDs for the bio-extraction some HOCs (Boucher et al., 2008). This study focused on the extraction of HOCs including atrazine and naphthalene, which have molecular weights ranged from 122 to 291 (g mol^-1^). These HOCs exhibit a low to moderate hydrophobicity (log *K*_ow_ 1.3–3.8) but are not regarded as highly toxic in comparison with POPs such as dioxins. Therefore, the main purpose of the current work was to study the physicochemical properties of LDs from date palm seeds and their applicability for extraction of POPs such as dioxins from polluted aqueous environments.

## Materials and Methods

### Plant Materials and Chemicals

Date palm (*Phoenix dactylifera* L.) seeds, also known as stones, were collected from fruits of the Sukary cultivar, imported from Kingdom of Saudi Arabia. Seeds were isolated, washed, air-dried, and stored in plastic bags at room temperature until required. To determine the effect of seed germination on the quantity and quality of LD fraction, seeds were germinated *in vitro* in a current of running water for 2 weeks before extracting LDs. The intumescent seeds were sown onto two layers of gauze and covered with two further layers of a solidified-plastic transparent box (20 cm × 13 cm × 8 cm). Culture boxes were placed in an incubator at 30 ± 2°C and humidified daily and seedlings were obtained 15 days after sowing. Seedlings with a radicle length of 0.5 or 2 cm were referred as stages I and II, respectively. Non-germinated seeds (stage 0) and germinated seeds at stages I and II were taken and subjected separately to the LD isolation as described below. 2,3,7,8-tetrachlorodibenzo-*p*-dioxin (2,3,7,8-TCDD dissolved in toluene at 10 μg mL^-1^, purity 99%) was purchased from Supelco, Inc., USA. Physicochemical properties of 2,3,7,8-TCDD are presented in Additional file 3 (**Supplementary Table S1**) From the commercial TCDD solution, supplied as a 1-mL ampule of TCDD at 10 μg mL^-1^, all of the TCDD was placed in a 10-mL capped glass tube and evaporated to dryness under nitrogen. For health and environmental safety reasons, residual TCDD was re-dissolved in a minimum volume (100 μL) of dimethyl sulfoxide (DMSO), and 5 mL of aqueous solutions of TCDD were prepared in deionized and distilled water to obtain initial concentrations of 0, 10, 50, and 100 ng L^-1^ TCDD. For treatment of seeds with TCDD, seeds were germinated as described above and humidified daily with the prepared solutions of TCDD at various concentrations. Seedlings at stages 0, I, II, and II were taken for further analysis.

### Isolation of LDs from Date Palm Seeds

Lipid droplets were isolated from seeds of date palm (*Phoenix dactylifera* L.) according to Hanano et al. (2006) with a few modifications. Due to their stony nature, seeds were germinated in running water for 2 weeks then subjected to dry grinding using a high performance grinder (Cross Beater Mill SK, Retsch, Germany) to produce a well-hulled and crushed grain. A brief sieving was used to separate the woody cover particles of the seeds from their stony core pieces (0.5 mm). Into a brass mortar, five grams of the ground stony core were taken and milled in liquid nitrogen until a fine powder was obtained. After the liquid nitrogen was evaporated, the powder (5 g) was hydrated with 10 mL of buffer A (100 mM potassium pyrophosphate, 0.1 M sucrose and pH 7.4). The mixture was gently homogenized for 5 min using an ultra-dispenser (T25 digital ULTRA-TURRAX, IKA Laboratory, Germany) and centrifuged for 10 min at 10,000 × *g*. The supernatant was subjected to a second centrifugation at 100,000 × *g* for 1 h and a floating white pad layer, consisting of LDs, collected from the top of the tube using a Pasteur pipette. LDs were carefully and sequentially washed twice with 5 mL of buffer B (buffer A without sucrose). After a final centrifugation (100,000 × *g* for 1 h), the LD fraction was suspended in 2 mL of buffer B and stored at 4°C for further analysis.

### Physicochemical Characterization of LDs

The amount of free fatty acids in isolated date palm oil bodies was determined by a colorimetric method using palmitic acid and oleic acid as standards (Nixon and Chan, 1997). Protein concentrations were estimated by the Bradford assay (Bradford, 1976). The water content of LD suspensions was determined gravimetrically. 0.2 ml of LD suspension was evaporated in a desiccator under vacuum to constant mass (24 h). Encapsulation of LDs was evaluated by a method based on extraction of lipids by hexane (Matsuno and Adachi, 1993). The amount of lipid extracted by hexane from the LD fraction is a measure of the success of the encapsulation. The smaller the amount of extractable lipid, the better the encapsulation. Aggregation and coalescence of LDs as a function of pH was performed by suspending the isolated LDs in buffer B at pH values of 2.5, 3.5, 4.5, 5.5, 6.5, and 7.5 and immediately analyzing them by light microscopy or after an incubation period of 12 h at room temperature. Proteolysis of LDs was carried out according to Hanano et al. (2006). Briefly, proteinase K 50 μg was added to 1 mL of LDs containing approximately 5 mg of associated proteins and the reaction mixture was incubated for 1 h at 37°C. After proteinase treatment, the stability and size of date palm LDs were examined by light microscopy and imaging.

### Light and Electronic Microscopy, Flow Cytometry, and Particle Size Determination

Lipid droplets were subjected to microscopic analysis directly after each extraction without making any suspension. LD suspensions were agitated in glass test tubes prior to measurements to ensure homogeneity and drops were placed on glass slides to observe for microscopy. Microscopic imaging was done at 40 and 100× using a LEICA MPS60 microscope and using an Olympus FE-4000 camera and with a scanning electron microscope (SEM; Vega II XMU, TESCAN, Czech Republic). Purity of LDs, their native encapsulation and their number per mL were evaluated by a Flow cytometry (BD FACSCALIBUR, Biosciences, USA). LD size distributions (% frequency) were determined using a laser granulometer (Malvern Mastersizer S; Malvern Instruments, England) fitted with a 320 mm lens as described (White et al., 2006).

### Protein Isolation from LDs

Lipid droplet-associated proteins were isolated according to the method by Katavic et al. (2006). Briefly, 0.5 mL hexane was mixed with 0.5 mL of isolated LDs and centrifuged for 5 min at 13.000 × *g*. After centrifugation, the interfacial layer and bottom aqueous phase were subjected to a flow of nitrogen gas to remove any remaining hexane. 0.75 mL chloroform/methanol (2:1, v/v) was mixed with the interfacial layer and centrifuged for 5 min at 13.000 × *g*. The protein-rich interfacial layer was taken into 0.25 mL water, mixed with 0.75 mL chloroform/methanol (2:1, v/v) and centrifuged for 5 min at 13,000 × *g*. The procedure was repeated twice. After washing, the LD-protein pellet was suspended in 0.5 mL water, precipitated in four volumes of cold 100% acetone for 16 h at -20°C and resuspended in buffer B.

### SDS-PAGE and Western Blotting

Proteins were analyzed by sodium dodecyl sulfate polyacrylamide gel electrophoresis (SDS-PAGE) using 12% polyacrylamide gels and electroblotted onto a PVDF membrane (Millipore) in a Semi-Dry Transfer Cell (Bio-Rad). Caleosins were immunodetected by incubating the membrane with a polyclonal antibody prepared from the complete sequence of the Clo1 caleosin isoform from *Arabidopsis thaliana*, as described by Hanano et al. (2006). The signal was detected in a Pharos FX molecular imager (Bio-Rad).

### Transcriptional Analysis by RT-qPCR

Changes in relative transcriptional abundance of genes encoding LD-associated proteins in response to TCDD exposure were analyzed by reverse-transcription quantitative PCR (RT-qPCR; Hu et al., 2009). Briefly, frozen fine powder (1 g) samples from whole seedlings at stages 0, I, and II were used to extract total RNA using an RNeasy kit according to the manufacturer’s instructions (Qiagen, Germany). The quality of extracted RNAs was checked on agarose gels and concentrations measured by a Nanodrop device (Nano Vue, GE Healthcare). Remaining traces of genomic DNA were digested by DNase I (Fermentas, USA) and the lack of trace genomic DNA in total RNA was confirmed by a control PCR using total RNA as the template. Aliquots of 1 μg total RNA were used for first-strand cDNA synthesis according to Hanano et al. (2014). Real-time PCR was performed as described by Hanano et al. (2014). In brief, 25 μL reaction mixtures contained 0.5 μM of each specific oligonucleotide primer for the target genes (*OLEO, CLO*, and *STEROLEO*) and the reference genes (*Actin-1 and Tubulin-β7*; Additional file 4, **Supplementary Table S2**), 12.5 μL of SYBR Green PCR mix (Bio-Rad, USA) and 100 ng cDNA. QPCR conditions were as described previously (Hanano et al., 2014). The relative expression of target genes was normalized using two reference genes *Act-1* and *Tub-β* (Hu et al., 2009). Each measurement was performed in triplicate together with a dilution series of the reference gene. PCR efficiencies were between 95 and 105% (data not shown) and the average of *C*_T_ was taken. The relative quantification RQ of target genes was calculated directly using the software from the qPCR system. Sequences of amplified regions were confirmed by sequencing on an ABI 310 Genetic Analyzer using a Big Dye Terminator kit (Applied Biosystems).

### Sorption of TCDD in Date Palm LDs

The sorption essay was carried out by a simplified method as described by Boucher et al. (2008). In brief, 0.1 g of LDs (*m*_LD_) was suspended in 1 mL of buffer B and gently added into a 15 mL polypropylene test tube containing 5 mL of TCDD solution at initial concentrations of 0, 10, 50, and 100 ng L^-1^. Two control tubes were produced, one without TCDD and another without LDs. From the water content measured in LD suspensions, a dilution factor (*d*) was calculated taking in consideration the additional dilution of the TCDD solution by the water from LD suspensions so that *V*_aq_ = [*V*_TCDD_ + (*d* × *V*_LD_)]. During sorption, tubes were agitated on a rotary shaker for 1–5 h and then centrifuged for 10 min at 100.000 × *g.* After sorption essay, the floated LD fraction and the aqueous phase were sampled and subjected to extraction and analysis for TCDD content. From the initial aqueous concentration of TCDD (*C*^0^_TCDD_,_aq_[ng L^-1^]) and the aqueous equilibrium concentration (*C*^∗^_TCDD_,_aq_ [ng L^-1^]), the corresponding equilibrium concentration of TCDD in the LD *C*^∗^_TCDD,LD_ [ng g^-1^LD] may be calculated from a simple mass balance as following: *C*^∗^_TCDD,LD_ = [(*V*_aq_/*m*_LD_) × (*C*^0^_TCDD_,_aq_ -*C*^∗^_TCDD_,_aq_)], where *V*_aq_ is the total aqueous volume (mL) and *m*_LD_ is the mass of LD (g). The partition coefficient *K*_LD/_*_w_*,_TCDD_ [L g^-1^] was determined by linear regression as *K*_LD/_*_w_*,_TCDD_ = (*C*^0^_TCDD_,_aq_/*C*^∗^_TCDD_,_aq_). For partitioning in oil (LLE), the partition coefficient *K*_oil_/_w,TCDD_ is without units as the concentrations of TCDD in the oil and in the aqueous phase are both expressed in ng L^-1^. A correction factor, *j*, was thus introduced in order to express the partition in LD as a function of the oil content of the LD suspension. This factor *j* is a measure of the oil content of the LD suspension [g L^-1^]. Therefore, the real partition coefficient of TCDD into LDs is *K*_oilLD/w,TCDD_ = *j*^∗^*K*_LD/w,TCDD_ [L g^-1^].

### Extraction and Purification of TCDD

The extraction and purification of TCDD were done as in Hanano et al. (2014) with minor modifications. Briefly, approximately 2 mL of aqueous phase or 1 mL of the LD layer were mixed with 2 mL of 37% HCl and 5 mL of 2-propanol, homogenized and then extracted with equal volume of hexane by shaking vigorously for 1 h. After a brief centrifugation, the organic phase was taken for a second extraction for 1 h. The combined extracts were evaporated to dryness and re-dissolved in 1 mL of hexane, acidified with 125 μL HCl (2 M) and then extracted twice with 2 mL of hexane. Extracts were cleaned up using a small column containing 0.5 g anhydrous Na_2_SO_4_ on top and 1.0 g of florisil at the bottom. This column was activated with 3 mL of dichloromethane (DCM)/hexane/methanol (50:45:5) and TCDD eluted with 5 mL DCM/hexane/methanol. The eluates were evaporated to dryness and dissolved in 100 μL hexane for GC/MS analysis. A spiked sample with a known concentration of TCDD (50 ng L^-1^) was used to validate the extraction and purification procedure.

### TCDD Analysis by HR-GC/MS

The 2,3,7,8-TCDD content was quantified by HR-GC/MS using an Agilent Technologies 7890 GC System (USA) coupled to an AMD 402 high resolution mass spectrometer (Germany) as described previously (Hanano et al., 2006). Details of the MS analysis and quality control are described in EPA methods 1613B and 1668A. A 1-μL aliquot of the sample was injected into an Agilent DB-5 MS fused silica capillary column (60 m × 250 μm ID, film thickness 0.25 μm) with helium as carrier gas at a constant flow rate of 1.6 mL min^-1^. The oven temperature program was as described by Shen et al. (2009) as follows: start at 150°C, hold for 1 min, increase to 200°C at 12°C min^-1^, increase to 235°C at 3°C min^-1^ and hold for 8 min, and finally increase to 290°C at 8°C min^-1^ and hold for 20 min. Quantification was performed using an isotope dilution method.

### Bioinformatic Methods

Oleosin-, caleosin-, and steroleosin-like sequences from the date palm genome were obtained from the National Center for Biotechnology Information (NCBI) database^1^. Protein names, NCBI-accession numbers, ordered loci, amino acid compositions, molecular weights and isoelectric points (pI) are shown in **Table 1**. To show the presence of potential LD-binding domains for oleosins, caleosins and steroleosins, hydrophobicity plots were created using the Kyte and Doolittle algorithm^2^ (Kyte and Doolittle, 1982) with a window size of 19 for each sequence as shown in Additional file 1, **Supplementary Figure S1**. Protein domains were analyzed via UniProt^3^. The LD protein sequences were analyzed for predicted proteinase K cleavage sites using http://web.expasy.org/peptide_cutter/. Multiple-sequence alignments of oleosin, caleosin, and steroleosin amino acid sequences were performed using Vector NTI Advance 11.5 software (Life Technologies, Carlsbad, CA, USA) and the Clustal Omega methods (Sievers et al., 2011). Phylogenetic analysis was carried out using the neighbor-joining method of Saitou and Nei (1987), and displayed using Phylogeny Analysis^4^.

**Table 1:** Details of genes encoding orthologs of oleosin-, caleosin-, and steroleosin-like proteins in the date palm genome.

Protein class	NCBI accession #	Order locus	Amino acids	MW (kDa)	Isoelectric point (pl)
**Oleosins**					
OLEO-like1_DATE	XP_008775902	LOC103696145	140	14.72	5.22
OLEO-like2_DATE	XP_008780784	LOC103700664	144	15.41	10.79
OLEO-like3_DATE	XP_008786272	LOC103704664	132	13.95	9.99
OLEO-like4_DATE	XP_008798606	LOC103713449	125	12.53	10.2
OLEO-like5_DATE	XP_008810983	LOC103722275	159	16.88	10.33
OLEO-like6_DATE	XP_008784976	LOC103703777	126	12.93	11.79
OLEO-like7_DATE	XP_008777688	LOC103697576	133	13.71	9.74
OLEO-like8_DATE	XP_008801946	LOC103715925	140	14.51	10
OLEO-like9_DATE	XP_008795990	LOC103711567	158	16.44	10.4
**Caleosins**					
CLO-like1_DATE	XP_008803896	LOC103717338	202	22.27	8.36
CLO-like2_DATE	XP_008775946	LOC103696186	239	26.49	8.35
CLO-like3_DATE	XP_008801250	LOC103715420	236	26.6	5.61
CLO-like4_DATE	XP_008775947	LOC103696190	175	19.26	5.95
CLO_like5_DATE	XP_008796441	LOC103711900	103	11.92	5.14
**Steroleosins**					
STEROLEO_Like1_DATE	XP_008811369	LOC103722549	330	37.09	5.92
STEROLEO_Like2_DATE	XP_008798974	LOC103713735	349	38.48	6.72
STEROLEO_Like3_DATE	XP_008806995	LOC103719497	336	37.84	6.20
STEROLEO_Like4_DATE	XP_008788738	LOC103706421	195	21.34	7.22
STEROLEO_Like5_DATE	XP_008798975	LOC103713735	256	28.38	7.56


### Statistical Analysis

All data are expressed as mean ± standard deviation (SD). Statistical analysis was performed using STATISTICA software, version10 (StatSoft, Inc.). Comparisons between control and treatments were evaluated by ANOVA analysis. Difference from control was considered significant as *P* < 0.05 or very significant as *P* < 0.01.

## Results

### Recovery and Physico-biochemical Characteristics of Date Palm LDs

**FIGURE 1:**
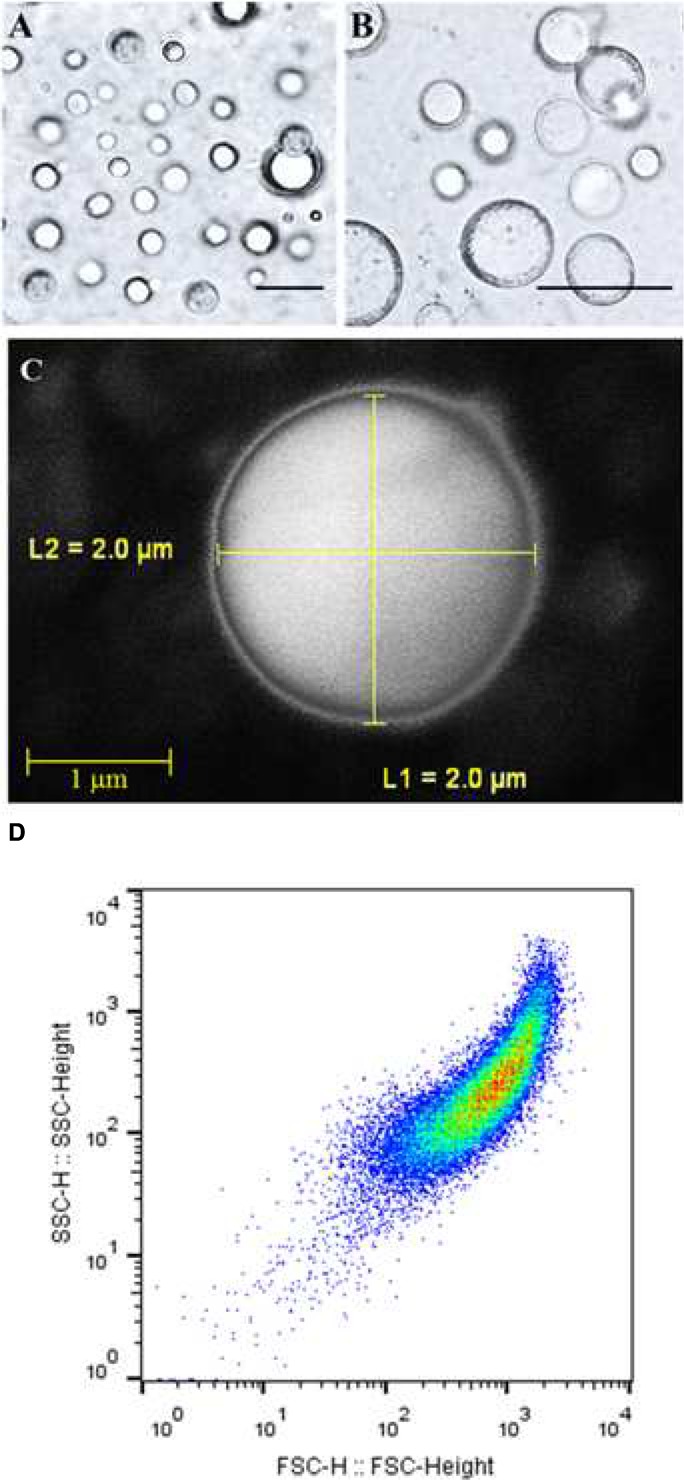
**Light and electronic micrographs and Flow Cytometer analysis of the isolated LDs from seeds of date palm.** **(A,B)** Recovered LDs suspended in 100 mM potassium pyrophosphate at pH 7.4 were observed under a LEICA MPS60 microscope and the images viewed at a magnification of 40 and 100×, respectively. Bar represents 5 mm. **(C)** The LDs were examined with a scanning electron microscope (SEM). **(D)** The purity, size, and number of date palm LDs were evaluated by a Flow Cytometer.

The isolated and purified LD fraction represented about 10% w/w of total seed weight. The LD fraction contained a total of 392.5 mg mL^-1^ lipid, 4.6 mg mL^-1^ protein, and 102.3 mg mL^-1^ water, respectively representing about 78.5, 1.05, and 20.4% of total amount of the LD fraction. In order to determine the native structures of isolated LDs, they were subjected to hexane extraction, a classical method to measure the success of an encapsulation state. This method showed that 81% of LDs in the preparation had an intact membrane. Light and scanning electron microscopic images showed the presence of spherical LDs with a clear surrounding membrane (**Figures 1A–C**). Moreover, the purity and the native structure of LDs were confirmed by flow cytometry and their number per mL was determined to be about 65.800 LD mL^-1^ (**Figure 1D**). Thus the LDs in these preparations can be considered as stable biological microcapsules.

### Aggregation and Coalescence of LDs as a Function of pH and Proteinase Treatment

**FIGURE 2:**
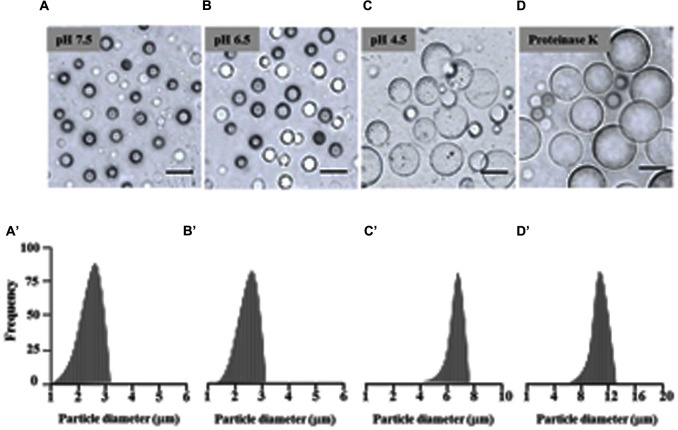
**Effects of pH and proteinase treatment on the aggregation/coalescence of date palm LDs.** Aggregation and coalescence of LDs as a function of pH was performed by suspending isolated LDs in buffer B (see Materials and Methods) at various pH 2.5, 3.5, 4.5, 5.5, 6.5, and 7.5 and immediately analyzed under light microscope or after an incubation for 12 h at room temperature. **(A–D)** Representative light micrographs of LDs suspended in pH 7.5, 6.5, 4.5 or after proteinase treatment, respectively. Images were obtained at a magnification of 40×. Bar represents 5 mm. **(A’–D’)** Size distributions (% frequency) of LDs in the different media at the indicated pH were determined using a laser granulometer as described in Section “Materials and Methods.”

There is evidence that isolated LDs aggregate when the pH of the suspension medium is reduced to below the isoelectric point of their major proteins (Tzen et al., 1992; Murphy, 1993). Therefore the effect of pH on date palm LDs was investigated. Of particular interest was the finding that the LDs did not aggregate in a medium of pH 7.5 and 6.5, even after the preparation had been left at 23°C for 12 h (**Figures 2A,B**). LD aggregation was observed when the pH was lowered to 5.5, with the maximum effect at pH 4.5 (**Figure 2C**). The prevention of aggregation (especially at pH 7.5 to 6.5) may be due to steric hindrance caused by the protruding N-terminal and C-terminal domains of proteins coating the surface of the droplets; indeed, removal of such domains by proteinase digestion is known to result in coalescence of isolated LDs (Tzen et al., 1992; Murphy, 1993). This was tested on date palm LDs and it was found that the coalescence of LDs was induced by proteinase K digestion at pH 7.5. After proteinase K treatment, LDs rapidly coalesced to form a milky layer at the top of the solution that was made up of much large diameter LDs (**Figure 2D**). We measured the size of LDs along the indicated pH range or after proteolysis, and found that the sizes of LDs were normally about of 2.2–2.5 m diameter at pH 7.5 and 6.5, respectively (**Figures 2A’,B’**). However, the LD diameters were doubled or tripled when the pH medium was lowered to 4.5 or after proteolysis (**Figures 2C’,D’**). This suggests that date palm LDs are normally very stable and their aggregation and/or coalescence do not occur unless they meet an acidic aqueous environment or their boundary proteins are cleaved by proteolysis.

### Proteomic Analysis of LD-Associated Proteins

**FIGURE 3:**
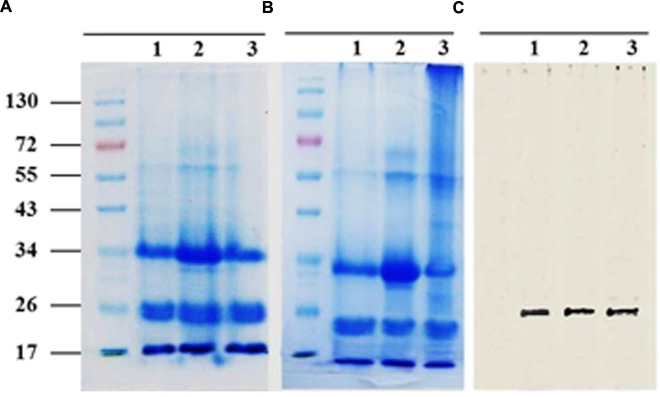
**Sodium dodecyl sulfate polyacrylamide gel electrophoresis (SDS-PAGE) analysis for LD-associated proteins of date palm.** Proteins were isolated from native or proteinase K-treated LDs as described in Section “Materials and Methods.” Resulted proteins from both samples of LDs were firstly quantified using protein assay from BioRad. **(A)** Three duplicate samples of 20 mg of native proteins and **(B)** Proteolytically treated proteins. Proteins were analyzed on 12% SDS-PAGE gels stained with G250 Coomassie blue. **(C)** Western immunoblot of caleosins detected by a polyclonal antibody prepared from the complete sequence of Clo1 caleosin from *Arabidopsis thaliana*, diluted 1:500 in TBS buffer (pH 7.4). A horseradish peroxidase-conjugated anti mouse IgG (Sigma-Aldrich, USA), diluted 1:2000, served as the secondary antibody. The signal was detected in a Pharos FX molecular imager (Bio-Rad; **C**, lanes 1–3). Five mL of a broad range protein marker was loaded to give bands at the indicated molecular weight in kDa.

Sodium dodecyl sulfate polyacrylamide gel electrophoresis analysis of solubilized LD-associated proteins showed three broad prominent bands with mean molecular masses of about 32–37, 22–24, and 17–20 kDa (**Figure 3A**). When bound to LDs, it is suggested that for thermodynamic reasons such proteins expose both their polar N- and C-terminal domains to the cytosol (Frandsen et al., 2001; Murphy, 2001). To experimentally test this, proteinase K-treated LDs were subjected to SDS-PAGE analysis. The LD-bound proteins were not completely degraded but their sizes were reduced by 2–5 kDa as shown in **Figure 3B**. Our bioinformatic analysis showed that the predicted date palm oleosin, caleosin and steroleosin proteins contained numerous proteinase K digestion sites throughout their sequences, which should have resulted in their complete hydrolysis by proteinase K (Supplementary Table S3). The finding that the proteins were only partially hydrolyzed is therefore consistent with the majority of each of these LD-associated proteins being embedded within the lipidic core of the LDs and therefore not accessible to proteinase K attack.

The presence of one of the most abundant classes of LDs-associated proteins, the caleosins, was confirmed by immunoblotting with a polyclonal antibody prepared from full length CLO1 from *Arabidopsis thaliana*. The CLO1 protein is a closely related ortholog of the predicted caleosins from the date palm genome. The anti-CLO1 antibody reacted positively to a protein band at about 26 kDa in three duplicate samples (**Figure 3C**). These data suggest that the mid-range group of date palm LD-associated proteins with relative masses of 22–24 kDa included members of the caleosin family.

### Partitioning and Absorption Kinetics of TCDD into Date Palm LDs

**FIGURE 4:**
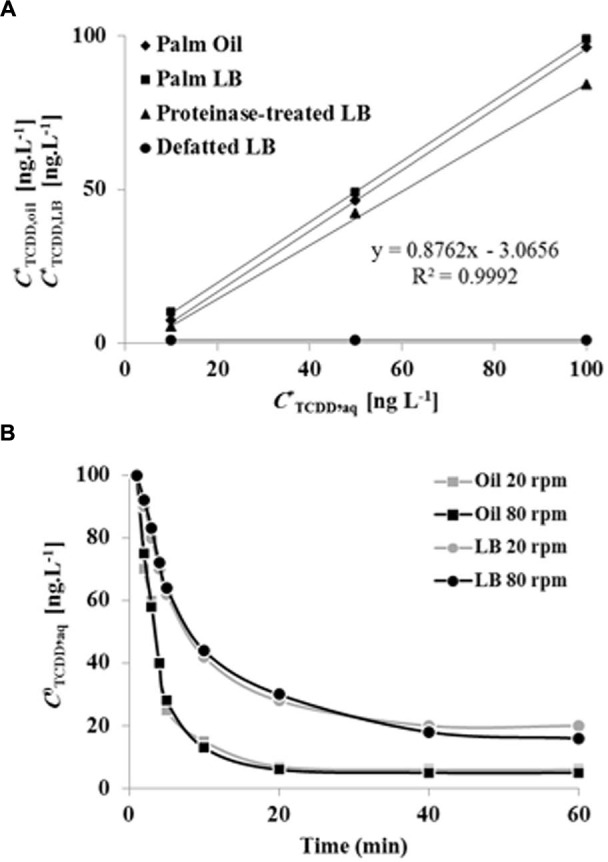
**Equilibrium plots and kinetics of TCDD partitioning into palm oil, date palm LDs, proteinase K-treated LDs and defatted LD.** **(A)** TCDD partitioning into palm oil (rhomboids), date palm LDs (squares), proteinase K-treated LDs (triangles) and defatted LDs (circles). For assays with oil or LDs, the total oil content was fixed at 5% [w/w] for a total volume of 6 mL. For defatted LDs, an equal volume of LDs was defatted with diethylether. The sorption assay was carried out on a rotary shaker at 240 rpm for 60 min in the presence of 0, 10, 50, and 100 ng L^-1^ TCDD. Extraction and analysis of TCDD was carried out as described in Section “Materials and Methods.” Three separated sorption experiments were performed for each concentration of TCDD and the subsequent analysis of extracted TCDD by HR-GC/MS was done in triplicate. **(B)** Kinetics of TCDD partitioning in palm oil or date palm LDs with shaking at 20 and 80 rpm. Measurements realized in (82 mm long, 16.8 diameter) rounded end polypropylene tubes, with a total volume in the tube of 6 mL. Three measurements were done for three individual experiments. Data are mean values ± SD (*n* = 6).

The absorption of TCDD into LDs was evaluated and compared with proteinase-treated LDs, defatted LDs, and with crude date palm oil. Due to the high values of the partition coefficients for TCDD into crude oil or LD fractions, we used the log value of the TCDD-partition coefficient to present the data. At equilibrium, the log of the partition coefficient for TCDD between the aqueous phase and the palm oil was determined to be log *K*_oil_/_w,TCDD_ = 7.462 ± 0.021 (**Figure 4A**). Further extractions were performed by replacing the crude oil by a given volume of LD suspension. This volume was calculated from the measured oil content of the LD suspension, in order to maintain a constant amount of oil (*m*_oil_) in all treatments. TCDD was effectively partitioned in the LDs with a partition coefficient of *K*_LD/w,TCDD_ = 5.205 ± 0.013 (L g^-1^). The value for the partition coefficient in the LD suspension is expected to be dependent on the oil content. Thus the partition coefficient was also expressed as a function of the oil content in the LD suspension, using correction with the factor *j*. In this way a value of log *K*_oilLD/w,TCDD_ = 7.528 ± 0.024 was obtained, which is very similar to the partition coefficient obtained for LLE in palm oil, as illustrated by the superimposed equilibrium lines (**Figure 4A**).

The results clearly showed that sorption of TCDD in LDs suspension occurred and that this sorption was dependent on the presence of oil within the LDs. This was further confirmed since defatted LDs showed essentially no sorption of TCDD. Sorption experiments using proteinase-treated LDs also yielded identical partition coefficients (**Figure 4A**). The kinetics of partitioning of TCDD into LDs was compared to partitioning into crude oil (LLE) under a given agitation. As shown in **Figure 4B**, TCDD partitioning was extremely fast in LDs (<10 min), even at the low agitation rate of 20 rpm, while approximately 30 min was required at a higher agitation rate (80 rpm) with palm oil (LLE). This demonstrates that TCDD is effectively partitioned into date palm LDs and, although it is essential for LD stability, the surface protein coating of LDs is not implicated in the partition mechanism for TCDD.

### TCDD-Removal Activity of LDs as a Function of pH

**FIGURE 5:**
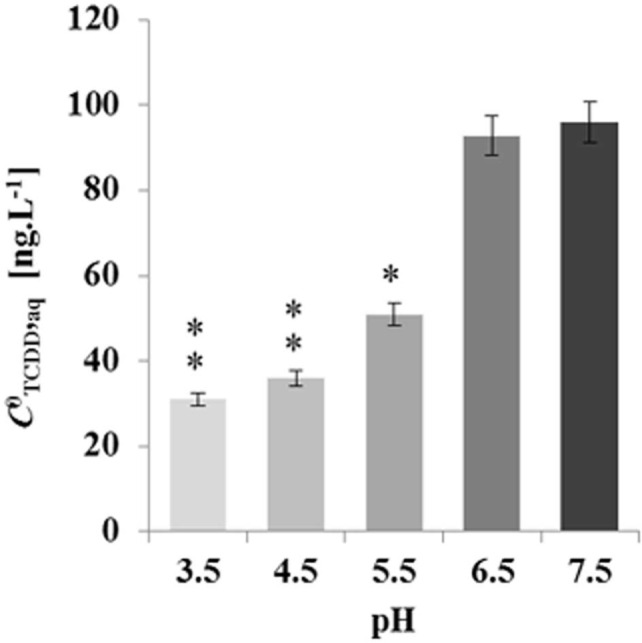
**Decreasing the absorption activity of date palm LDs to TCDD as a function of the pH medium.** TCDD [*C*^0^_TCDD_,_aq_ (100 ng L^-1^)] was partitioned into LDs in suspension medium at various pH 3.5, 4.5, 5.5, 6.5, and 7.5. The sorption assay was carried out on a rotary shaker at 240 rpm for 60 min in the presence of 100 ng L^-1^ TCDD. Extraction and analysis of TCDD was performed as described in Section “Materials and Methods.” Three separated sorption experiments were done and the subsequent analysis of extracted TCDD by HR-GC/MS was done in triplicate. Data are mean values ± SD (*n* = 6). Statistical significance was evaluated by ANOVA analysis. Asterisks indicate significant differences between absorption at neutral and acidic media: ^∗^*P* < 0.05 (significant); ^∗∗^*P* < 0.01 (very significant).

To elucidate whether the pH-effect on the spherical structure of LDs might have a subsequent effect on LD bioaffinity toward TCDD, we evaluated the sorption of TCDD into date palm LDs as a function of the pH of the medium. The data in **Figure 5** show that about 95% of TCDD [*C*^0^_TCDD_,_aq_ (100 ng L^-1^)] was partitioned into LDs when the pH of the suspension medium was 7.5 and 6.5. This declined to about 62, 41, and 36% at pH 5.5, 4.5 and 3.5, which indicates that the pH of the suspension medium influences the affinity of TCDD to LDs, and when the pH is decreased the absorption is reduced.

### Effect of Germination on LD Quality and Their Subsequent Bioaffinity Toward TCDD

**FIGURE 6:**
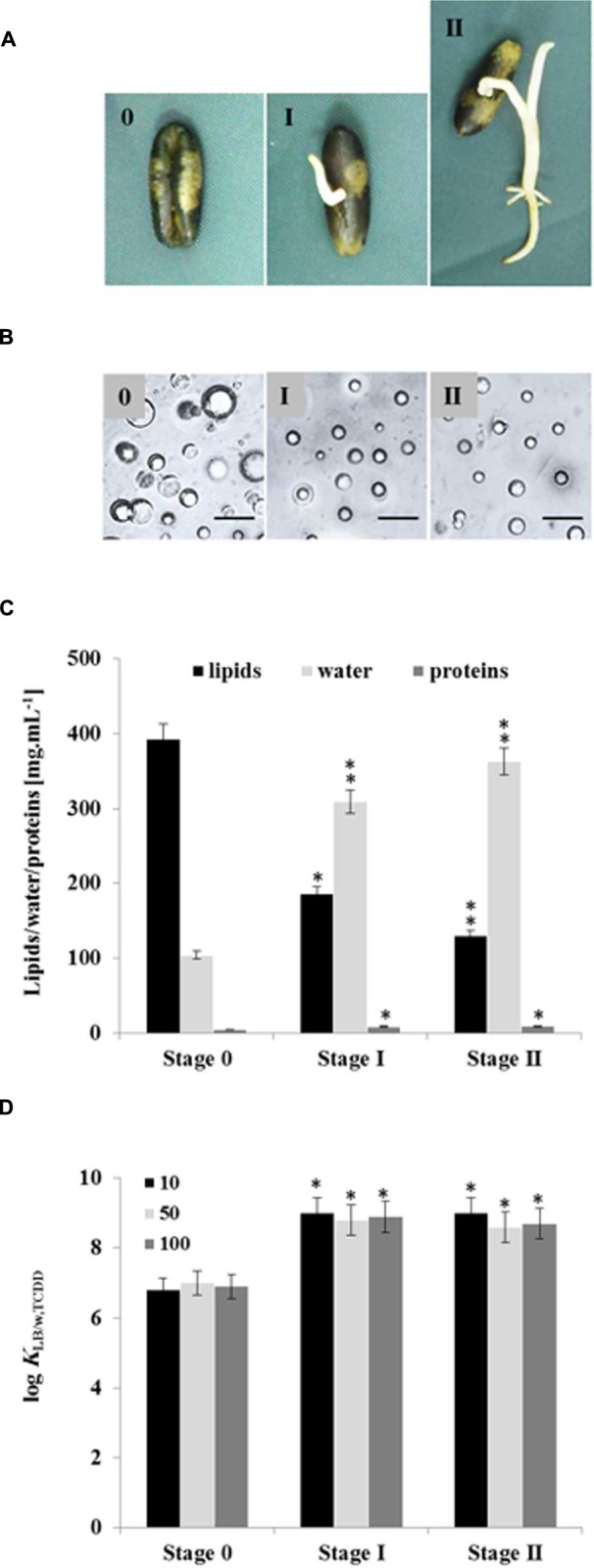
**Quantitative and qualitative effects of germination on seed LDs and their subsequent bioaffinity toward TCDD.** **(A)** Seedlings with a radicle length of 0.5 or 2 cm were referred as stages I and II, respectively, were compared with non-germinated seeds (stage 0). **(B)** Light micrographs of the isolated LDs from stages 0, I, and II. Recovered LDs suspended in 100 mM potassium pyrophosphate at pH 7.4 were observed under a LEICA MPS60 microscope and the images taken at magnification of 40×. Bar represents 5 mm. **(C)** Biochemical composition of the recovered LDs from indicated stages. Analysis of lipids, water and proteins contents were done in triplicate. **(D)** TCDD-partitioning into LDs isolated form stages 0, I, and II. Three separated sorption experiments were carried out and the subsequent analysis of extracted TCDD by HR-GC/MS was done in triplicate. Thus the partition coefficient was expressed as a function of the lipid content in the LDs of stages I and II. Data are mean values ± SD (*n* = 6). Statistical significance was evaluated by ANOVA analysis. Asterisks indicate significant differences between stage 0 and stages I and II: ^∗^*P* < 0.05 (significant); ^∗∗^*P* < 0.01 (very significant).

Seed LDs are mobilized as an energy source by the plant during germination with resultant changes in their lipid and protein contents (Huang, 1992; Murphy, 1993, 2001, 2012). We therefore investigated whether LDs isolated from germinated seeds at stages I and II had different bioaffinities toward TCDD compared with LDs from non-germinated seeds (**Figure 6A**). Under the microscope, the average diameters of LDs isolated from seedlings in stages I and II were smaller (≈1 m) than LDs isolated from seeds (stage 0; ≈2.5 m; **Figure 6B**). Enrichment of preparations in LDs occurred as a function of germination stage and LD recovery expressed as a dry weight was lower in stages I and II by factors of 1.6 and 2.2×, respectively when compared with stage 0 (data not shown). Moreover, the lipid content decreased in the LD fraction isolated from stages I and II by factor of 2 and 3, respectively compared with stage 0.

Conversely, the proportion of proteins in the LD fractions was doubled in both stages I and II (**Figure 6C**). As pointed out previously, the value for the partition coefficient of TCDD in the LD suspension is dependent on the lipid content. Thus when the partition coefficient was expressed as a function of the lipid content in the LDs of stages I and II, mean values of log *K*_LDstageI/w,TCDD_ = 9.918 ± 0.041 and *K*_LDstageII/w,TCDD_ = 8.768 ± 0.036 were obtained, which are higher than the partition coefficient obtained for LDs of stage 0 (*K*_LDstage0/w,TCDD_ = 7.218 ± 0.028), as illustrated in **Figure 6D**. The results clearly show that: (i) the recovery and the size of LDs are lowered in germinated seeds, (ii) they have lower contents of lipids and more of LD-associated proteins, (iii) the partition coefficient of TCDD between the aqueous phase and LDs from stages I and II is higher than its value for LDs from stage 0.

### Bioinformatic Analysis of Date Palm Putative LD-Associated Proteins

**FIGURE 7:**
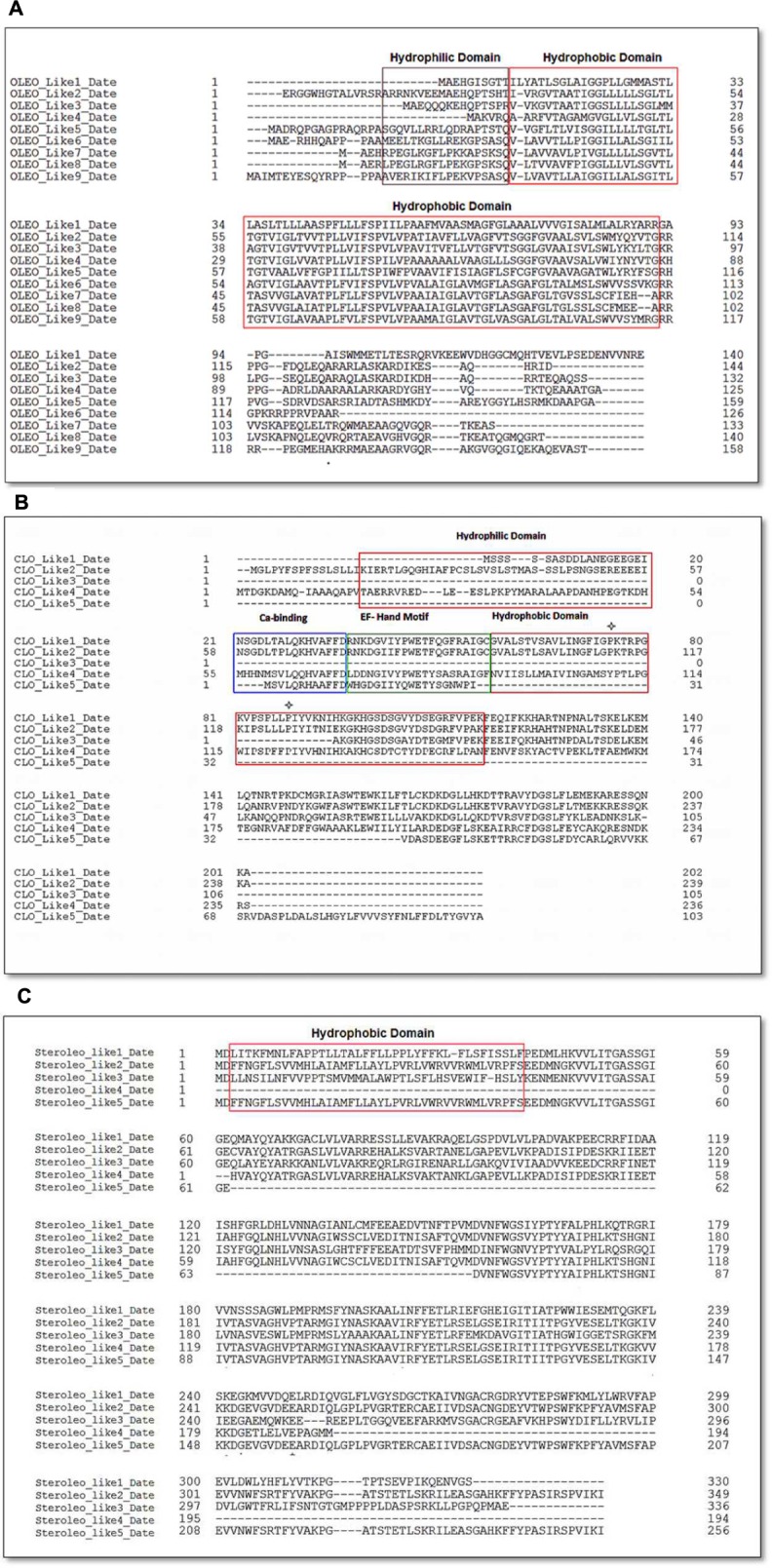
**Amino acid sequence alignments of date palm oleosins **(A)**, caleosins **(B)**, and **(C)** steroleosins.** Multiple sequence alignments were performed using Clustal omega. Protein domains properties were analyzed using Uniprot platform (http://www.uniprot.org). **(A)** Amino acid sequence alignment of date palm for oleosins. Domain colors are red for hydrophobic and brown for hydrophilic domains. **(B)** Amino acid sequence alignment for caleosins. Domain colors are red for hydrophobic, brown for hydrophilic domains, green for EF hand motifs and blue for Ca^2+^ binding domains. The ^∗^ notation shows the proline knot motif that starts from PK and ends at LLP. **(C)** Amino acid sequences alignment for steroleosins. Domain colors are red for hydrophobic and brown for hydrophilic domains.

Availability of the complete genomic sequence of date palm (Al-Mssallem et al., 2013) enabled us to carry out a detailed bioinformatic analysis of the genes and the putative encoded proteins that fall into the three major classes of LD-associated proteins, namely oleosins, caleosins, and steroleosins (Vance and Huang, 1987; Naested et al., 2000; Lin et al., 2002; Jolivet et al., 2004; Umate, 2012; Huang and Huang, 2015). For the most abundant LD-associated protein family, the oleosins, nine orthologs were found in the date palm genome with predicted masses in the expected range of 13–17 kDa. These are termed OLEO-like1_DATE to OLEO-like9_DATE in **Table 1**. As shown in **Figure 7A**, in all cases the predicted oleosins contained the three canonical oleosin domains reported from other plant species, namely an N-terminal amphipathic domain, a central hydrophobic LD-anchoring region, and a C-terminal amphipathic α-helical domain (Lin et al., 2002; Umate, 2012; Huang and Huang, 2015). In particular, the date palm oleosins had a highly conserved central hydrophobic domain with the proline knot motif of 12 residues (PXXXXXSPXXXP) that is known to be essential for stabilization of LDs both *in vivo* and *in vitro* (Huang and Huang, 2015).

In the case of caleosins, five potential orthologs were detected, referred as CLO-like1_DATE to CLO-like5_DATE in **Table 1**. As with the oleosins, the predicted size range of four of these proteins, namely 19–26 kDa, was consistent with published results from other plants and with our immunoblotting analysis (**Figure 3C**). In one case, namely CLO-like5_DATE, the predicted protein was significantly truncated compared to the other caleosins and while it had a predicted LD-binding domain, several other canonical caleosin domains were missing (**Figure 7B**). Therefore, while it seems unlikely that this ortholog has the capacity for the range of enzymatic functions of previously described caleosins, it might still function as a structural component of date palm LDs. The remaining four caleosins of date palm contained the four canonical structural features of previously described plant caleosins, namely; a 12-residue EF-hand motif involved in Ca^2+^-binding, a central hydrophobic domain including a proline knot (PXXXPSPXXP), two histidine residues responsible for iron binding (heme axial ligand), and a serine kinase phosphorylation site near the C-terminal (**Figure 7B**).

For the steroleosin group, the date palm genome contained five putative orthologs, shown as STEROLEO_Like1_DATE to STEROLEO_Like5_DATE in **Table 1**. The proteins predicted from first three steroleosin-like sequences were similar in mass to previously described members of this family of plant LD-associated proteins (Umate, 2012; Huang and Huang, 2015). However, the last two sequences were significantly shorter and lacked some structural motifs of other plant steroleosins (**Figure 7C**), although all five steroleosin-like sequences contained sufficiently long hydrophobic domains to enable them to bind to LDs (Additional file 1, **Supplementary Figure S1**).

### Transcriptional Analysis of Genes Encoding LD-Associated Proteins Following Exposure of Date Palm Seedlings to TCDD

**FIGURE 8:**
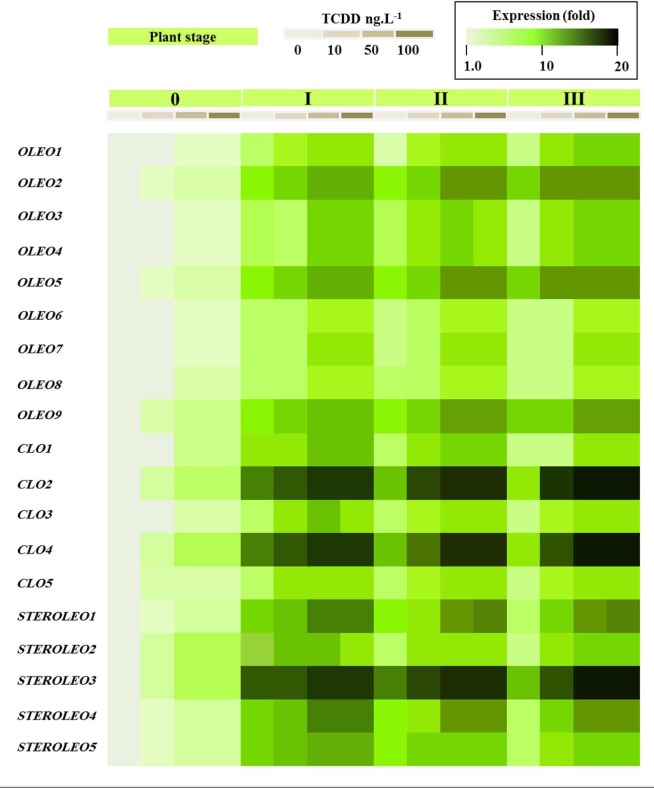
**Transcriptional analysis of the relative expression levels of oleosin, caleosin and steroleosins encoding genes (*OLEO*, *COL*, and *STEROLEO*) in whole date palm seedlings after exposure to TCDD (0, 10, 50, and 100 ng L^-1^).** For each gene, the transcript level was evaluated by qRT-PCR as described in Section “Materials and Methods.” Three measurements were taken in three cDNAs prepared from three individual plants for each treatment. The color scale (white-green-black) indicates relative changes of transcript abundance of 1, 10, and 20 fold, respectively. For each gene, the expression levels in seeds and seedlings of date palm unexposed to TCDD were defined as 1, and the corresponding abundance changes under 10, 50, and 100 ng L^-1^ TCDD were calculated directly using the software installed in the Applied Biosystems qPCR system.

**Figure 8** shows that all of the putative LD-related genes were expressed in seedlings but that their expression levels differed dramatically between different members of each gene family according to developmental stage and exposure to TCDD. In the case of the oleosins, three genes (*OLEO_like2, OLEO_like5*, and *OLEO_like9*) were expressed at significantly higher levels than other members of the gene family at stages I–III of seedling development and there was some up-regulation in response to TCDD exposure. In the case of the caleosins, two genes (*CLO_like2* and *CLO_like4*) were very highly expressed at stages I–III of seedling development and showed little response to TCDD exposure. Other members of the caleosin gene family showed more modest levels of expression but in some cases were up-regulated by as much as 10- to 15-fold in response to TCDD exposure. Finally, in the steroleosin gene family, one gene (*STEROLEO_like3*) showed very high expression levels at stages I–III of seedling development and two other gene family members (*STEROLEO_like1* and *STEROLEO_like4)* were up-regulated by as much as 15-fold in response to TCDD exposure.

## Discussion

This study reports the isolation, characterization, and deployment of date palm LDs as part of a possible broader biomimetic approach for removal of highly toxic organic hydrophobic pollutants, such as dioxins, from the environment. We use the term ‘biomimetic’ in view of a recently published study that described for the first time the presence of naturally occurring mechanism in fungi whereby LDs were shown to serve as sites for the sequestering of various types of lipophilic toxin (Chang et al., 2015). As discussed below, this appears to be part of a wider detoxification role for LDs that may be present in a wide range of other species, including bacteria, algae, and maybe animals (Gwak et al., 2014). Therefore we suggest that the strategy developed here for using plant LDs to sequester dioxins can be categorized as a biomimetic approach, whereby a naturally occurring biological function can be exploited for technological purposes. In the present case the main technological use for the LDs would be for environmental remediation following pollution by hydrophobic toxins such as dioxins.

Because dioxins are regarded as the most toxic group of so-called POPs, the physiochemical properties of LDs isolated from seeds of date palm (*Phoenix dactylifera* L.) were assessed with particular emphasis on their bioabsorption activity toward such pollutants. Seed LDs of many plants have been isolated (Vance and Huang, 1987; Huang, 1992, 1996; Leprince et al., 1998; Besbes et al., 2004; Katavic et al., 2006; Abdalla et al., 2012; Chapman et al., 2012; Walther and Farese, 2012; Al-Mssallem et al., 2013) but, to our knowledge, this is the first study of LDs from date palm seeds. Although, date palm seeds have lower oil contents compared with major oilseed crops (Besbes et al., 2004), the isolated oil fraction was highly pure in its LD content. The enrichment of LDs was comparable to those prepared from other seeds such as soybean, rice, maize and rapeseed (Sarmiento et al., 1997; Lin et al., 2002; Sandermann, 2003; Jolivet et al., 2004; Kelley et al., 2015).

Hexane extraction and microscopy imaging showed that the purified LDs in the initial buffer preparation (pH 7.4) were spherical and surrounded by an intact membrane, with an estimated mean diameter of about 2.2 m. This size is comparable to that recorded for plant LDs from other seeds (Huang, 1992; Chapman et al., 2012; Murphy, 2012). Moreover, our data showed that LDs of date palm conserve their individual spherical structures in neutral or slightly acidic pH, but that they aggregate and subsequently coalescence in acidic medium (pH 5.5). At physiological pH, LDs individually float in the cell during seed imbibition and their coalescence is prevented by the action of LD-associated proteins, especially oleosins (Murphy, 1993; Kelley et al., 2015). Tzen et al. (1993) have suggested that lowering of the pH of LD suspensions from 7.2 to about 6.0 further protonates the histidine residues and neutralizes the LD surface. As a result, aggregation occurs as confirmed by our findings where proteinase K-treated palm LDs coalesced and formed much larger diameter particles than the native LDs.

The aggregation/coalescence of LDs in a medium with a pH lower than their isoelectric point seems to be a universal physiochemical phenomenon (Murphy, 1993; Kelley et al., 2015). However, the onset of aggregation/coalescence varied according to the nature of LDs, to their native size, and to the biochemical composition of the terminal parts of proteins exposed on the surface of LDs (Tzen et al., 1992; Sarmiento et al., 1997; Lin et al., 2002; Kelley et al., 2015; Wahlroos et al., 2015). Previous studies have shown that LDs from a range of plant species have an isoelectric point in the range of pH 7.4–6.2 (Tzen et al., 1990, 1993; Murphy, 2012), contrary to our own measurement of pH 5.5. The tendency of date palm LDs to keep their native structural properties in neutral to slightly acidic solutions seems to be a valuable criterion that reinforces their potential for environmental uses. Proteomic analysis showed that the proteins isolated from the LDs of date palm were separated into three major bands of approximately 32–37, 20–24, and 16–18 kDa. These data are consistent with other findings where proteins with similar molecular weights were identified in LDs of various plants (Lu et al., 2006). The integration of these three protein groups into the LD membrane was confirmed by subjecting of LDs to a proteinase K-treatment. This eliminates any possible contamination with a similar proteins located in endoplasmic reticulum or within LDs (Murphy, 2001).

The affinity of dioxins to lipids is well-studied in mammals (Rifkind et al., 1990; Lawrence and Kerkvliet, 1998) but less so in plants (Hanano et al., 2014, 2015a). However, the affinity of dioxins to fractioned plant LDs in the aqueous phase has never been elucidated. Our results clearly show that efficient sorption of TCDD into LD suspensions occurred and that this sorption depended on the presence of TAG within the LDs since defatted LDs showed essentially no sorption of TCDD. To date, no comparative data exist about *in vitro* affinity of dioxins to plant LDs. It has been documented that HOCs show differential lipo-affinity toward TAGs or phospholipids (Sandermann, 2003). It seems that highly hydrophobic compounds (log *K*_ow_ > 3) show a higher affinity for TAG and vice-versa. Thus the high hydrophobicity known for TCDD (log *K*_ow_ = 6.8) can explain the high affinity of TCDD to the TAG-matrix LDs.

In line with this, Boucher et al. (2008) have studied the partitioning of atrazine into proteolysed LDs and found that the partitioning of this herbicide into normal LDs or proteolysed LDs did not change. It is interesting here to note that atrazine is a water-soluble herbicide and does not need a highly effective emulsifying activity to be absorbed into LDs. Such emulsifying activity is normally ensured by the exposed domains of proteins in the surface of LDs. In the present study with TCDD, which is known for its high hydrophobicity, it was found that its partitioning into normal or proteolysis LDs isolated from non-exposed seedlings to TCDD also did not change *in vitro*, a similar result to that obtained by Boucher et al. (2008). However, when seeds were germinated in the presence of TCDD, they formed smaller LDs with more of proteins compared with control and it appears that this increase was in favor of the functional proteins, caleosins and steroleosins rather than the structural proteins, the oleosins. Interestingly the partitioning of TCDD into these LDs was higher than in the control LDs. This indicates that the LDs-associated proteins can exhibit different influences, effects or roles regarding the interaction of LDs with external agents. In favor of this hypothesis, two points can be made: (i) the catalytic domain of such functional proteins is normally located inside the LDs core and proteolysis of the surface-located domains does not affect their enzymatic activity (Hanano et al., 2006). (ii) Any increase in the amount of lipid-metabolizing enzymes in LDs could possibly result in a modification to the biochemical and physicochemical nature of the TAG core favoring a ready access to external molecules.

A particularly critical point is the permeation of hydrophobic compounds through the monolayer membrane that surrounds LDs. In this regard, Boucher et al. (2008) showed that such compounds were absorbed into the inner TAG core of LDs and the monolayer membrane did not represent a barrier for the mass transfer of the compounds toward the TAG core. It has also been suggested that some compounds could form complexes with the phospholipids and/or proteins and adsorb onto the surface of biological membranes, thereby interfering with entry into the vesicle under the driving force of the TAG core (Banerjee et al., 1999).

Since lipid reserves are used as a source of energy during seed germination in date palm, we examined the composition of LDs and their subsequent affinity toward TCDD during the germination process. In accordance with previous findings, our data showed that the recovery of LDs was affected after seed germination and their lipids and proteins were highly modified. However, no alteration was observed in the integrity of remaining LDs. In this context, it was suggested that LDs in germinating seeds are not degraded simultaneously. More likely, glyoxysomes, with the assistance of mitochondria, fuse and digest LDs one at a time, while the remaining LDs are preserved intact during the whole period of germination (Tzen et al., 1997). Although, a gradual degradation of specific oleosins has been described during imbibition in maize (Bowman et al., 1988), the increasing amount of the proteins in LD fractions isolated from seedlings can be explained by the fact that the lipid-metabolizing enzymes become associated with LDs, caleosins and seed-specific lipoxygenase during seed germination (Tzen et al., 1997; Feussner et al., 2001). Supporting evidence on the role of oleosins in maintaining the biological structure of LDs comes from experiments where oleosin expression was suppressed in *Arabidopsis* whereby oleosin-deficient plants accumulated large LDs with a much reduced amount of coating proteins (Siloto et al., 2006). It was speculated that the large LDs would be generated by a number of coalescence events due to insufficient oleosin coverage on the surface of LDs (Siloto et al., 2006). Of a particular interest, increasing protein coating of LDs would increase of surface-to-volume ratio (Tzen et al., 1993; Murphy, 2012). The maintenance of high surface-to-volume ratios could facilitate access by TCDD by providing a high interaction surface between LDs and TCDD. Interestingly, our data showed that the partition coefficient of TCDD between the aqueous phase and LDs of seedlings was similar to with its value for seed LDs. This implies that for the same lipid content, small LDs absorb more TCDD than large LDs.

Computational analysis showed that the genome of date palm (*Phoenix dactylifera* L.) contained nine oleosin, five caleosin and five steroleosin putative orthologs. The phylogenetic analysis showed that date palm oleosins were subdivided into four well-supported groups (Additional file 2, **Supplementary Figure S2**). Despite the presence of the two oleosin isoforms (L-oleosin and H-oleosin) in many plant species (Gasteiger et al., 2005), palm have been found to contain only L-oleosins suggesting that L-oleosin is a primitive isoform, and H-oleosins is derived from L-oleosins (Tzen et al., 1990; Tai et al., 2002). The date palm transcriptomic data (**Figure 8**) showed that all of the analyzed LD-related genes were expressed during seedling development. However, certain members of each gene family were particularly highly expressed, most notably *CLO2*, *CLO4* and *STEROLEO3*, each of which displayed expression levels as much as 100-fold higher than other members of their respective gene families. Interestingly some members of each gene family also showed upregulation of expression in response to TCDD exposure. We have recently shown that in *Arabidopsis* plants exposed to TCDD, although overall seed oil content was reduced, the activity of some lipid metabolizing genes such as lipoxygenases was stimulated (Hanano et al., 2015a). Lipoxygenases are involved in the formation of oxylipins from TAG and other lipids and some of these oxylipins act as signaling molecules in various processes ranging from plant development to responses to stress (Hanano et al., 2015a). Caleosins have been shown to act as peroxygenases and to be involved in oxylipin metabolism, a variety of developmental processes, and a range of biotic and abiotic stress responses in both plants and fungi (Partridge and Murphy, 2009; Aubert et al., 2010; Blee et al., 2014; Shimada et al., 2014; Hanano et al., 2015b).

The overall concept of deploying LDs as agents to sequester lipophilic toxins has recently received validation from the natural world with reports that several fungal species employ cytosolic LDs for both defensive and aggressive purposes (Chang et al., 2015). In one case, the endolichenic fungus, *Phaeosphaeria* sp. manufactured plastoquinone derivatives that are normally phototoxic, but when these compounds were sequestered into LDs their toxicity was highly reduced. In other cases, cells of the yeast species, *Candida albicans* and *Saccharomyces cerevisiae*, which contained elevated levels of LDs, manifested greatly enhanced resistance to phototoxicity by the plastoquinone derivative, hypocrellin A. These LDs acted as sites for the safe sequestration of hypocrellin A, in a location where it was no longer capable of generating harmful reactive oxidative species (ROS; Chang et al., 2015). These LD-rich fungi were also able to sequester externally applied toxic lipophilic agents, such as farnesol, amphotericin and micronazole, in a way that protected them from oxidative damage. The protective effect of LDs in fungi was limited to lipophilic toxins and the LDs were ineffective against more polar antibiotic agents, such as terbinafine or caspofungin (Chang et al., 2015). In general, larger fungal LDs were more effective protective agents than small LDs as they were able to sequester larger quantities of the lipophilic toxins. Interestingly, the most effective protective activity was found in fungal LDs with a size range of about 1–2 μm, which is similar to the most abundant size range in the date palm LDs used in the present study.

In addition to these fungal studies, there have also been numerous reports of the protective roles of LDs in many plants and animals and an increase in LD accumulation is often found as part of their responses to various environmental stresses (Murphy, 2012). One example is the recent observation that macrozooid cells from the alga, *Haematococcus pluvialis* responded to the presence of ROS caused by photooxidative stress by upregulating LD accumulation and increasing the sequestration of lipophilic antioxidants that served to scavenge the ROS (Gwak et al., 2014). In other cases, mammalian LDs have been shown to act as sinks for the sequestration of lipotoxic agents such as non-esterified fatty acids and several classes of potentially disruptive proteins (Van Herpen and Schrauwen-Hinderling, 2008; Nguyen and Nosanchuk, 2011; Li et al., 2014). The use of LDs as temporary stores of potentially disruptive proteins has been also observed in several animal species (Li et al., 2014) and this mechanism is often hijacked by viruses in order to store and assemble viral coat proteins as these pathogens replicate within cells (Camus et al., 2013; Filipe and McLauchlan, 2015; Welte, 2015). This raises the possibility that suitably engineered LDs could also be used as binding sites to remove specific classes of proteins from aqueous mixtures.

## Conclusion

We suggest that our observation of the ability of date palm LDs to take up and remove highly potent environmental toxins, such as dioxins, from external media can be regarded as a biomimetic approach that could be used more generally as part of environmental remediation and/or purification strategies. It is noted that many organisms including plants, algae, fungi, and animals, have already evolved the ability to use endogenous LDs as part of a mechanism to sequester and detoxify a wide range of potentially harmful lipophilic agents from simple organic molecules to large proteins. This applies to both internally produced and externally generated toxic agents. It therefore appears that both internal and external detoxification of potentially harmful compounds constitutes yet another function of cellular LDs and that this could be used more broadly for biotechnological applications, especially in environmental remediation.

## Author Contributions

AH, led the work, designed all experiments, and wrote the manuscript. IA and MS carried out all experimental work. FR, performed the bioinformatics analysis. EB, read and commented the manuscript. DM, designed all computational analysis and edited all the body of the manuscript. AH and DM carried out revisions to the manuscript. All authors read and approved the final manuscript.

## Conflict of Interest Statement

The authors declare that the research was conducted in the absence of any commercial or financial relationships that could be construed as a potential conflict of interest.

## Footnotes

1 http://www.ncbi.nlm.nih.gov

2 http://www.ncbi.nlm.nih.gov/pubmed/7108955

3 http://www.uniprot.org

4 http://www.phylogeny.fr/simple_phylogeny.cgi

## Abbreviations

CLO: caleosin
HOCs: hydrophobic organic compounds
HR-GC/MS: high resolution-gas chromatography/mass spectrometry
LDs: lipid droplets
LLE: liquid–liquid extraction
OLEO: oleosin
PCDDs: polychlorinated dibenzo-p-dioxins
PCDFs: polychlorinated dibenzofurans
PLs: phospholipids
POPs: persistent organic pollutants
STEROLEO: steroleosin
TCDD: 2,3,7,8-polychlorinateddibenzo-p-dioxins
TAGs: triacylglycerols
