# Supplementary material for: Biochemical, Transcriptional, and Bioinformatic Analysis of Lipid Droplets from Seeds of Date Palm (Phoenix dactylifera L.) and Their Use as Potent Sequestration Agents against the Toxic Pollutant, 2,3,7,8-Tetrachlorinated Dibenzo-p-Dioxin
Source: Front Plant Sci. 2016 Jun 8;7:836. doi: 10.3389/fpls.2016.00836 (PMC4896926; doi:10.3389/fpls.2016.00836)
Supplement: Supplementary file 1 [file Data_Sheet_1.DOCX]

**Supplementary Materials and Mthods**

**Production of polyclonal anti CLO1-antibody in rabbit.**

Caleosin 1 (CLO1) (NCBI-accession number AEE85016) from Arabidopsis was previously cloned, expressed in *S. cerevisiae* and purified by affinity chromatography Hanano et al., JBC, 2066). All procedures and protocols used and the care of experimentation animals are performed according to the specifications established in Real Decreto 21/10/2005, on "Protection of animals used for experimentation and other scientific purposes”. Two mg of purified recombinant protein was used to produced protein antibody according to a **Standard Protocol for the Generation of Rabbit Polyclonal Sera at** <http://www.proteinalternatives.com/en/polyclonal_antibody_production.cfm>**.**

**Supplementary Figures**

**Additional file 1. Fig. S1.** Hydropathy plots of major lipid droplet proteins (for methods, see refs 41, 43)

OLEO-like 1


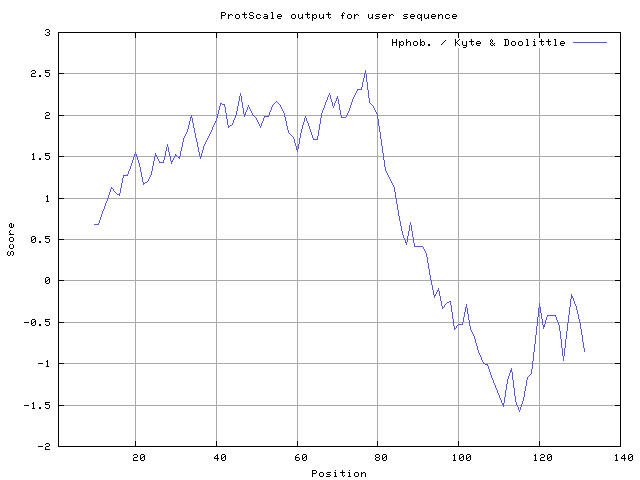


OLEO-like 2

**
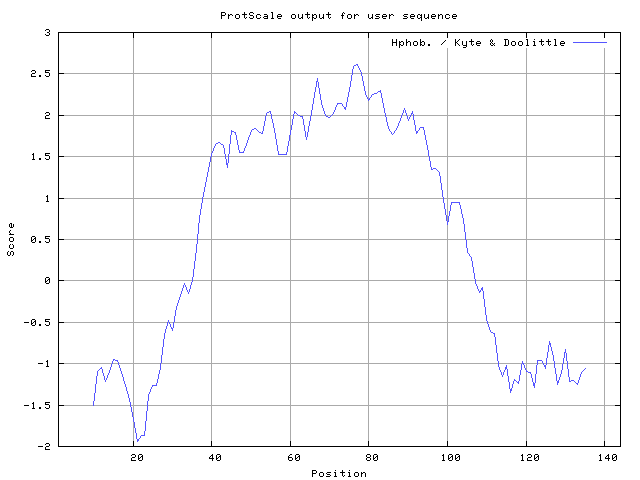
**

OLEO-like3


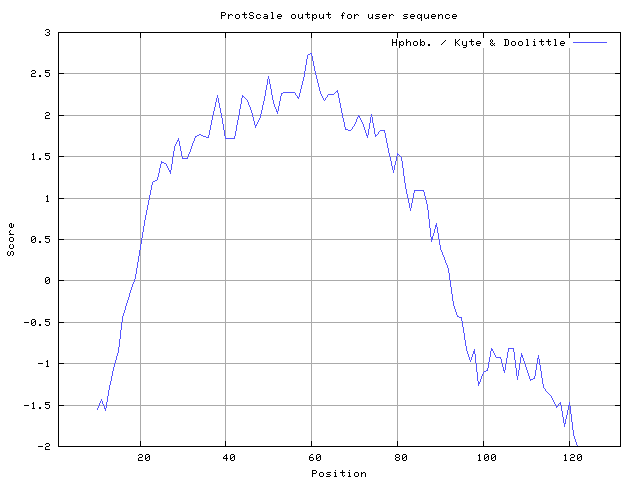


OLEO-like 4


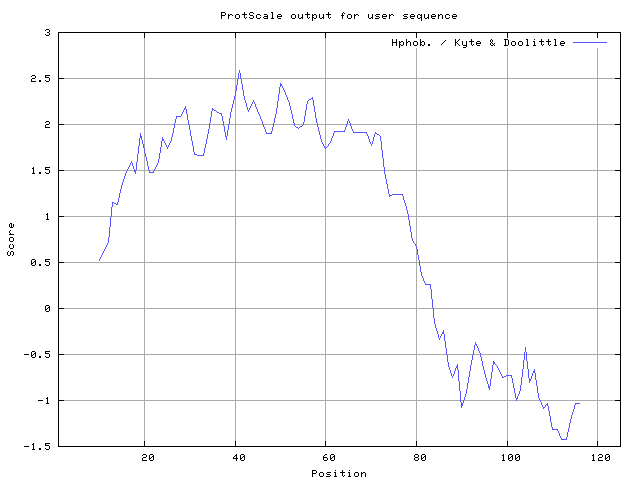


OLEO-like 5


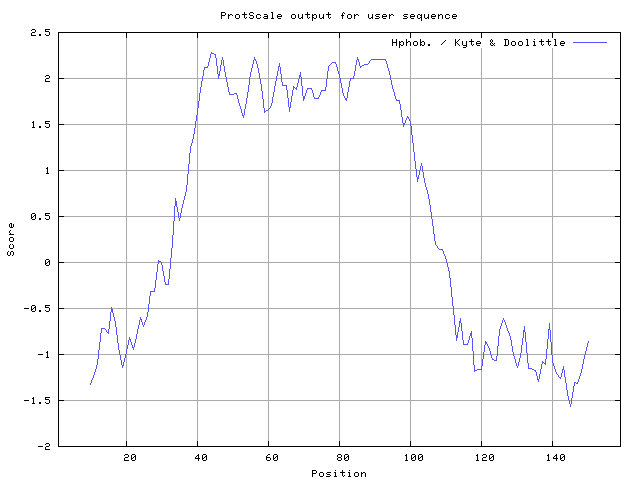


OLEO-like 6


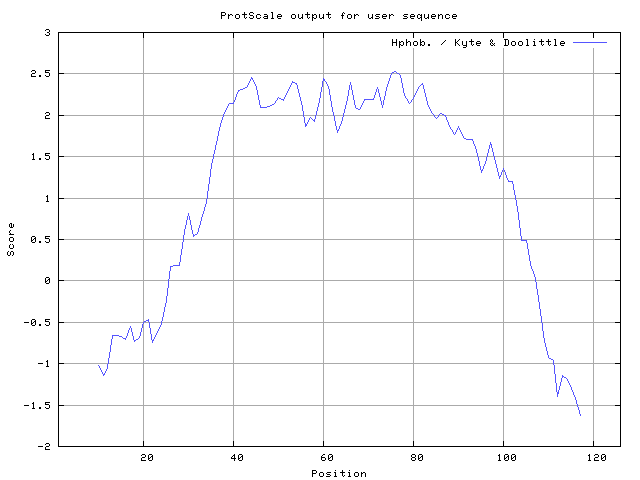


OLEO-like 7


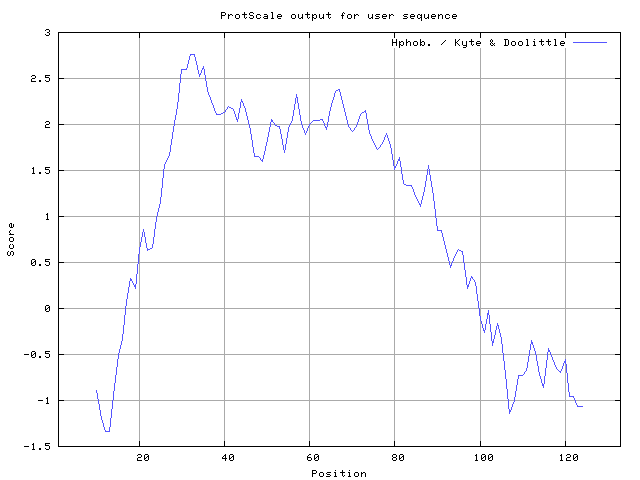


OLEO-like 8


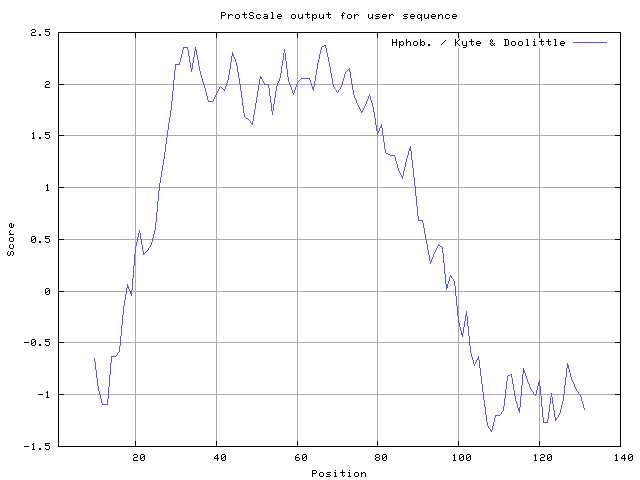


OLEO-like 9


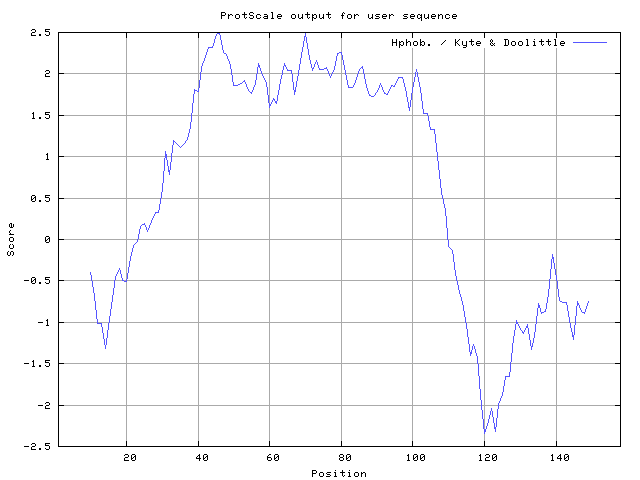


CLO-like 1


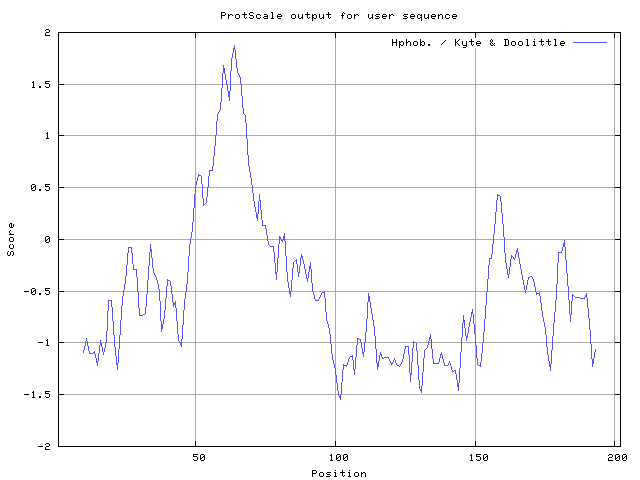


CLO-like 2


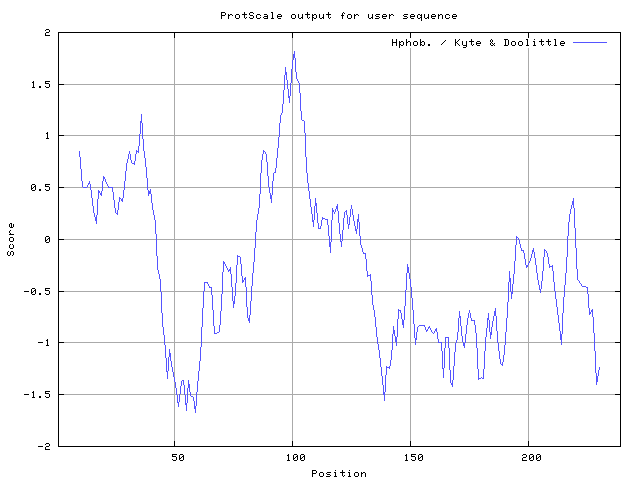


CLO-like 3


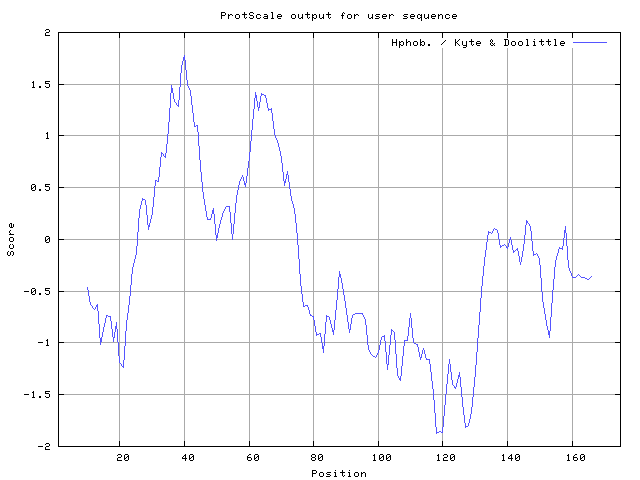


CLO-like 4


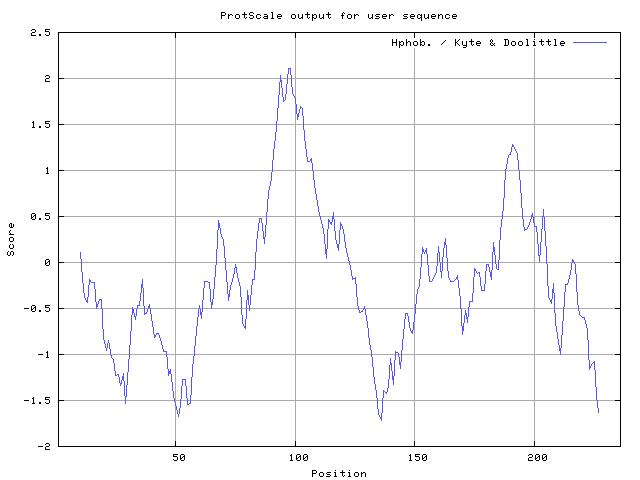


CLO-like 5


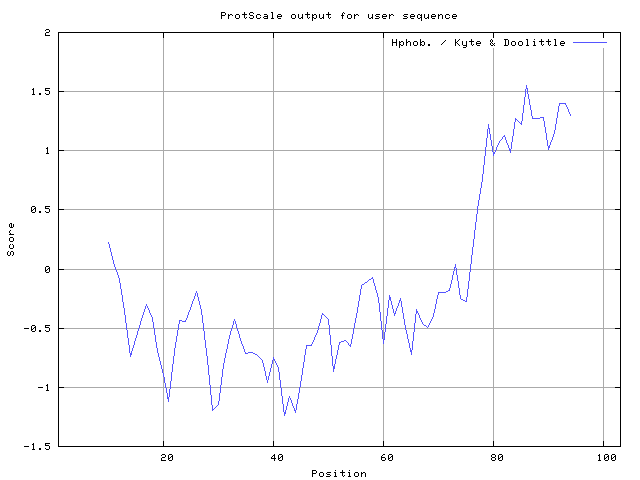


STER-like 1


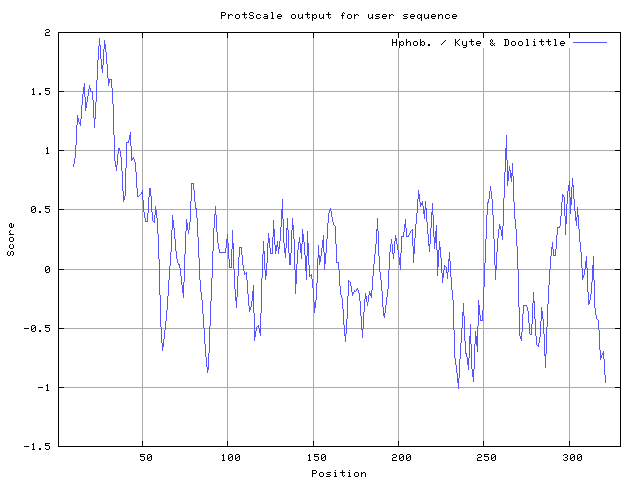


STER-like 2


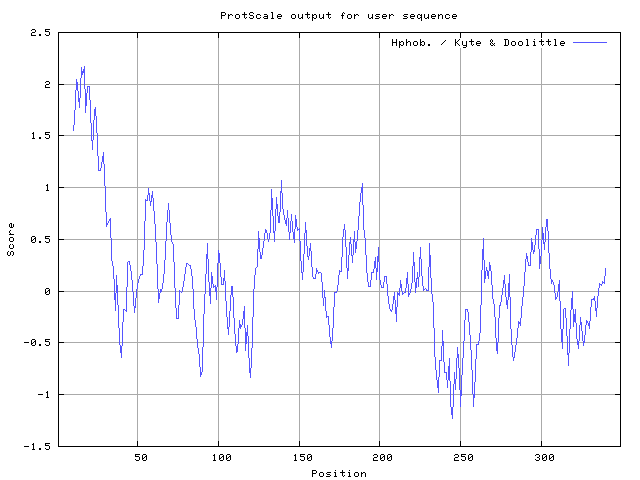


STER-like 3


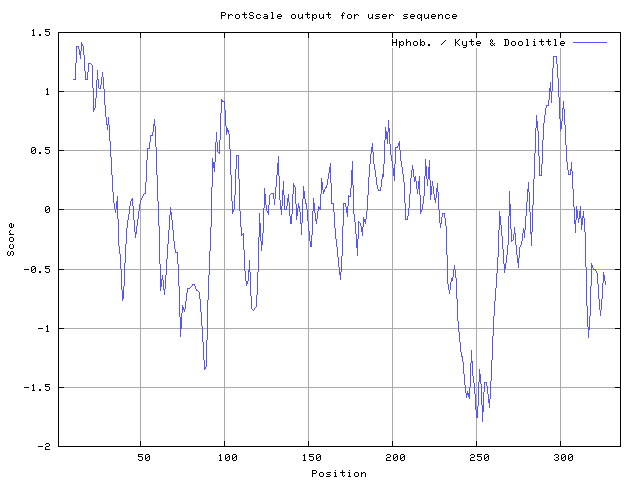


STER-like 4


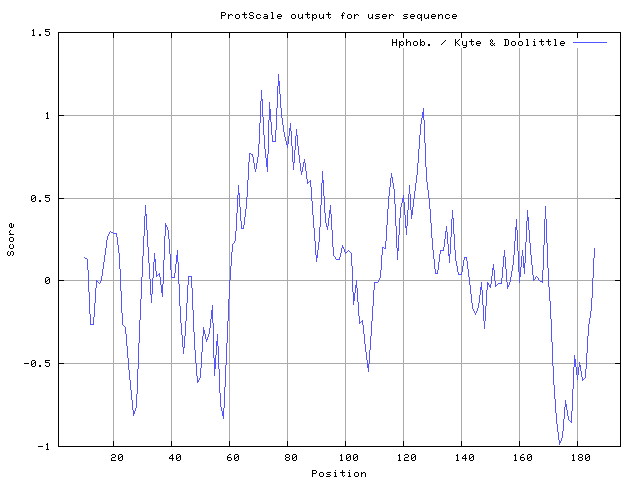


STER-like 5


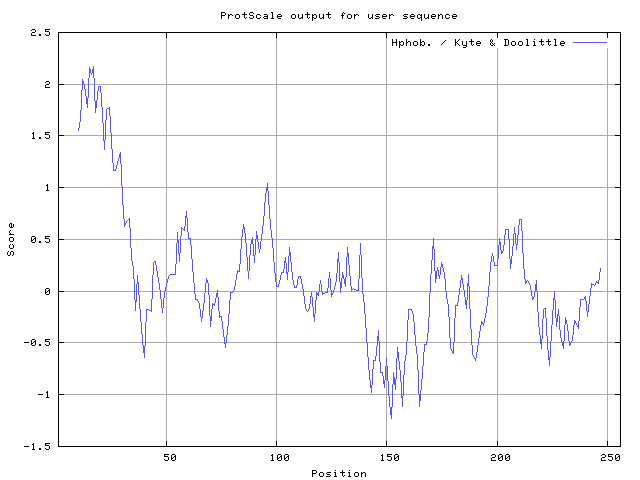


**Additional file 2. Fig. S2.** Phylogenetic relationship between the major LD-associated proteins in date palm. Phylogenetic trees were constructed using an alignment of full-length protein sequences identified from the date palm genome data. Phylogenetic analysis was carried out using the neighbour-joining method of Saitou and Nei (9), and the phylogenetic tree was displayed using Phylogeny Analysis online service (<http://www.phylogeny.fr/simple_phylogeny.cgi>).

1. Oleosin-like proteins


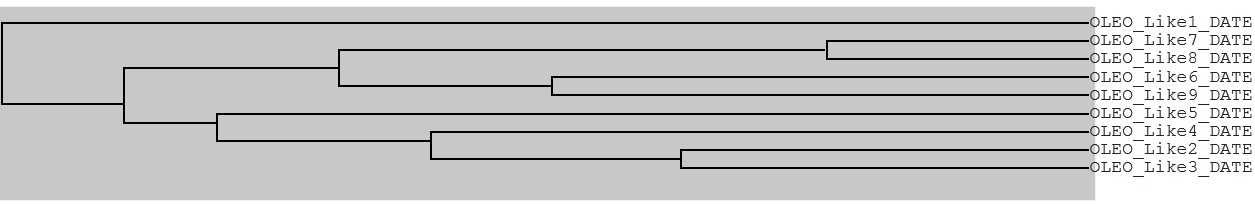


1. Caleosin-like proteins

**
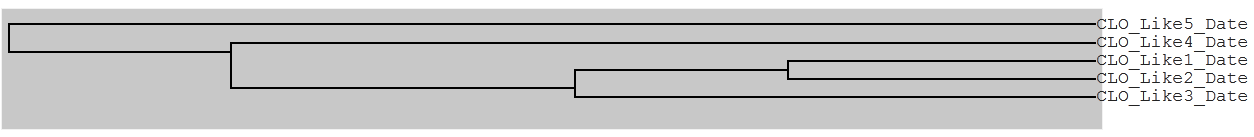
**

1. Steroleosin-like proteins


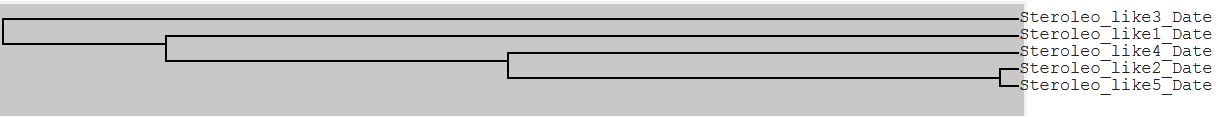


**Supplementary Tables**

**Additional file 3.** Table S1 Physicochemical properties of 2,3,7,8-tetrachlorodibenzo-*p*-dioxin (TCDD)

| Properties | Description/Value |
| --- | --- |
| Molecular Weight | 321.97 g mol^-1^ |
| Color/ Form/Odor | White crystalline needles |
| Vapor Pressure | 7.4x10-4 mm Hg, 25 C |
| Octanol/Water Partition (*K*_ow_): | Log *K*_ow_ = 6.8 |
| Water Solubility | 19.3 ng L^-1^ of water at 25 C; Insoluble in water |
| Soil Sorption Coefficient | *K*_oc_ N/A; very low mobility in soil |

**Additional file 4** Table S2 Primers used in this study

| Target gene | Order Locus | Forward/Revers Primers (5'-3') | Primers positions | Amplicon (bp) |
| --- | --- | --- | --- | --- |
| *OLEO-like1_DATE* | LOC103696145 | CAGCCCCATAATCCTCCCAG | 165-184 | 124 |
|  |  | CCTTCGGGCATAACGGAGAG | 288-269 |  |
| *OLEO-like2_DATE* | LOC103700664 | ATAAACGAATCACCGCCCCC | 455-474 | 137 |
|  |  | AAAAGGCGACCCACAAGACA | 591-572 |  |
| *OLEO-like3_DATE* | LOC103704664 | TCCTGCGTCTTCTTGGTCAT | 557-576 | 130 |
|  |  | CGCACAAACAGTAACCGCAA | 686-667 |  |
| *OLEO-like4_DATE* | LOC103713449 | CCACTACGTGCAGACCAAGA | 347-633 | 143 |
|  |  | CGGAATCACAAGCCCTACCA | 489-470 |  |
| *OLEO-like5_DATE* | LOC103722275 | TGCGTGCCATGAGTTAGTGT | 626-645 | 128 |
|  |  | ACTGCAGCGTTTGATCTCCA | 753-734 |  |
| *OLEO-like6_DATE* | LOC103703777 | GCTGATGTCACTCTCGTGGG | 327-346 | 91 |
|  |  | GACTTAACGGGCAGCTGGAA | 417-398 |  |
| *OLEO-like7_DATE* | LOC103697576 | CAGGGCAGGAGGTGAAAACA | 446-465 | 113 |
|  |  | CCCTGCGCAGCAACTTACTA | 558-539 |  |
| *OLEO-like8_DATE* | LOC103715925 | AGGCTCGTATCAAAGGCACC | 363-382 | 122 |
|  |  | TTTCATGTCCTGCCCTGCAT | 484-465 |  |
| *OLEO-like9_DATE* | LOC103711567 | CCAGGAGAAAGCCCAAGAGG | 480-499 | 149 |
|  |  | CCGACCAAGAACTGAAGCGA | 628-609 |  |
| *CLO-like1_DATE* | LOC103717338 | GGCTACCTCCACTGCTTTCT | 70-89 | 116 |
|  |  | CAGTCAGGTCGCCACTGTTT | 185-166 |  |
| *CLO-like2_DATE* | LOC103696186 | GGCGTCCTCATCGTTACCTT | 175-194 | 106 |
|  |  | GTTCCGGTCAAAGAAGGCGA | 280-261 |  |
| *CLO-like3_DATE* | LOC103696190 | ACCCGACAGAGACCTACGAA | 59-78 | 125 |
|  |  | GATTGCCGGTAGGCTTCCAT | 183-164 |  |
| *CLO-like4_DATE* | LOC103715420 | AGGCTATCAGGCGTTGCTTT | 681-700 | 106 |
|  |  | AGCCAAGGATTCGAGCAGTC | 786-767 |  |
| *CLO_like5_DATE* | LOC103711900 | AGCGACATGCTGCCTTCTTT | 14-33 | 140 |
|  |  | ATCAAAACAACGCCTGGTGG | 153-134 |  |
| *STEROLEO_Like1_DATE* | LOC103722549 | GTGGCAAGGAGAGAGAGCAG | 229-248 | 125 |
|  |  | GCATCGATGAACCTCCGACA | 353-334 |  |
| *STEROLEO_Like2_DATE* | LOC103713735 | CCGGCATTGGTGAGGATGTA | 189-208 | 131 |
|  |  | TCTTGCGGTAGGAACATGGC | 319-300 |  |
| *STEROLEO_Like3_DATE* | LOC103719497 | TCCCACGTATGTTGCCCTTC | 568-587 | 119 |
|  |  | TCCCGACAGCATCTGCATAC | 686-667 |  |
| *STEROLEO_Like4_DATE* | LOC103706421 | GGCACCGGAGGTTCTACTGA | 102-121 | 138 |
|  |  | ACGAGGCAGCTACACCAAAT | 239-220 |  |
| *STEROLEO_Like5_DATE* | LOC103713735 | CATTGGTGAGTGCGTTGCTT | 223-242 | 112 |
|  |  | CCCAAGCTCATTTGCAGTCC | 334-315 |  |
| *Actin-1* | AT2G37620 | CGGTATTGTGTTGGACTCTGG | - | 98 |
|  |  | CAGCAAGGTCAAGACGGAGT |  |  |
| *Tubulin-β7* | AT2G29550 | GAGTGGAGTTACCTGCTGCCT  ATGTAGACGAGGGAACGGAA |  | 94 |
|  |  |  | - |  |
